# 

**Published:** 1962-01

**Authors:** 


W1
ME359

### THE MEDICAL JOURNAL OF THE SOUTH-WEST

*Incorporating*
*The Bristol Medico-Chirurgical Journal*

NATIONAL
FEB 1962
MEDICINE

*January 1962*

VOL. 77 (i). No. 283

